# Supplementary material for: Rituximab-IgG2 is a phagocytic enhancer in antibody-based immunotherapy of B-cell lymphoma by altering CD47 expression
Source: Front Immunol. 2024 Dec 6;15:1483617. doi: 10.3389/fimmu.2024.1483617 (PMC11659266; doi:10.3389/fimmu.2024.1483617)
Supplement: Supplementary file 1 [file DataSheet1.pdf]

Rituximab-IgG2 is a phagocytic enhancer in antibody-based immunotherapy of B-cell lymphoma by altering CD47 expression

Oanh T.P. Nguyen[1], Sandra Lara[1], Giovanni Ferro[1], Matthias Peipp[2], Sandra Kleinau[1*]

[1]Microbiology and Immunology Program, Department of Cell and Molecular Biology, Uppsala University, Uppsala, Sweden

[2]Division of Antibody-Based Immunotherapy, University Hospital Schleswig-Holstein, Kiel, Germany

Supplementary Material

Supplementary Figures

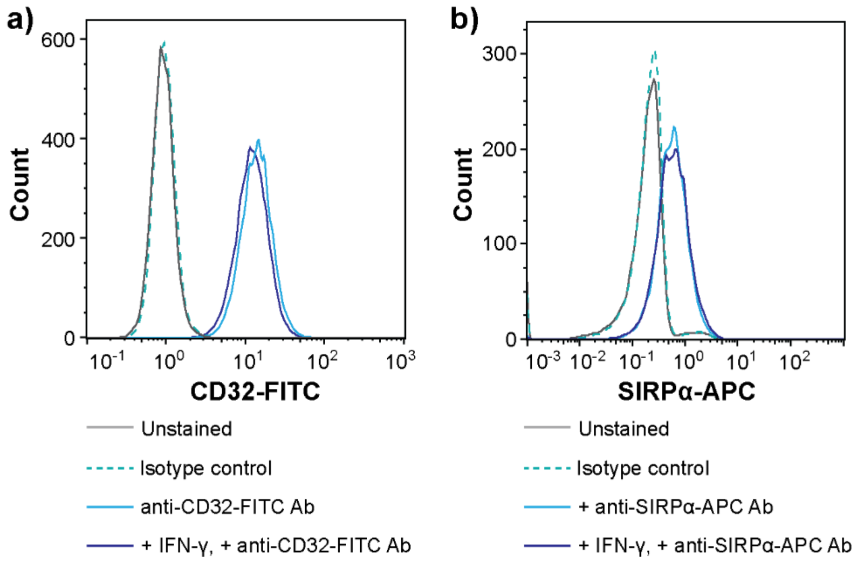

Figure S1. Representative histogram of a) CD32 and b) SIRP-α staining of unstimulated and IFNγ-stimulated MonoMac-6 cells.

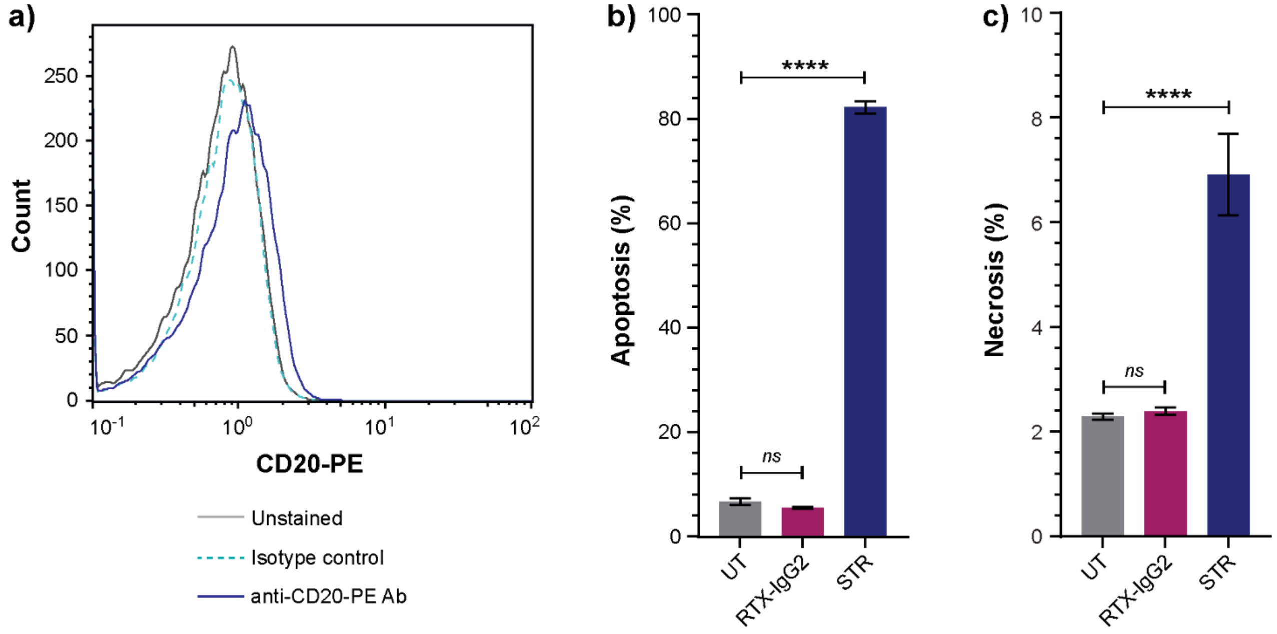

Figure S2. a) Representative histogram of CD20 staining of Reh cells. Analysis of b) apoptosis and c) necrosis in untreated (UT), RTX-IgG2 or STR-treated $CD20^{-}$ Reh cells. Data are presented as mean ± SEM of three biological replicates. Statistical analysis by one-way ANOVA with Tukey-Kramer post-hoc test (****: p<0.0001, ns = not significant).

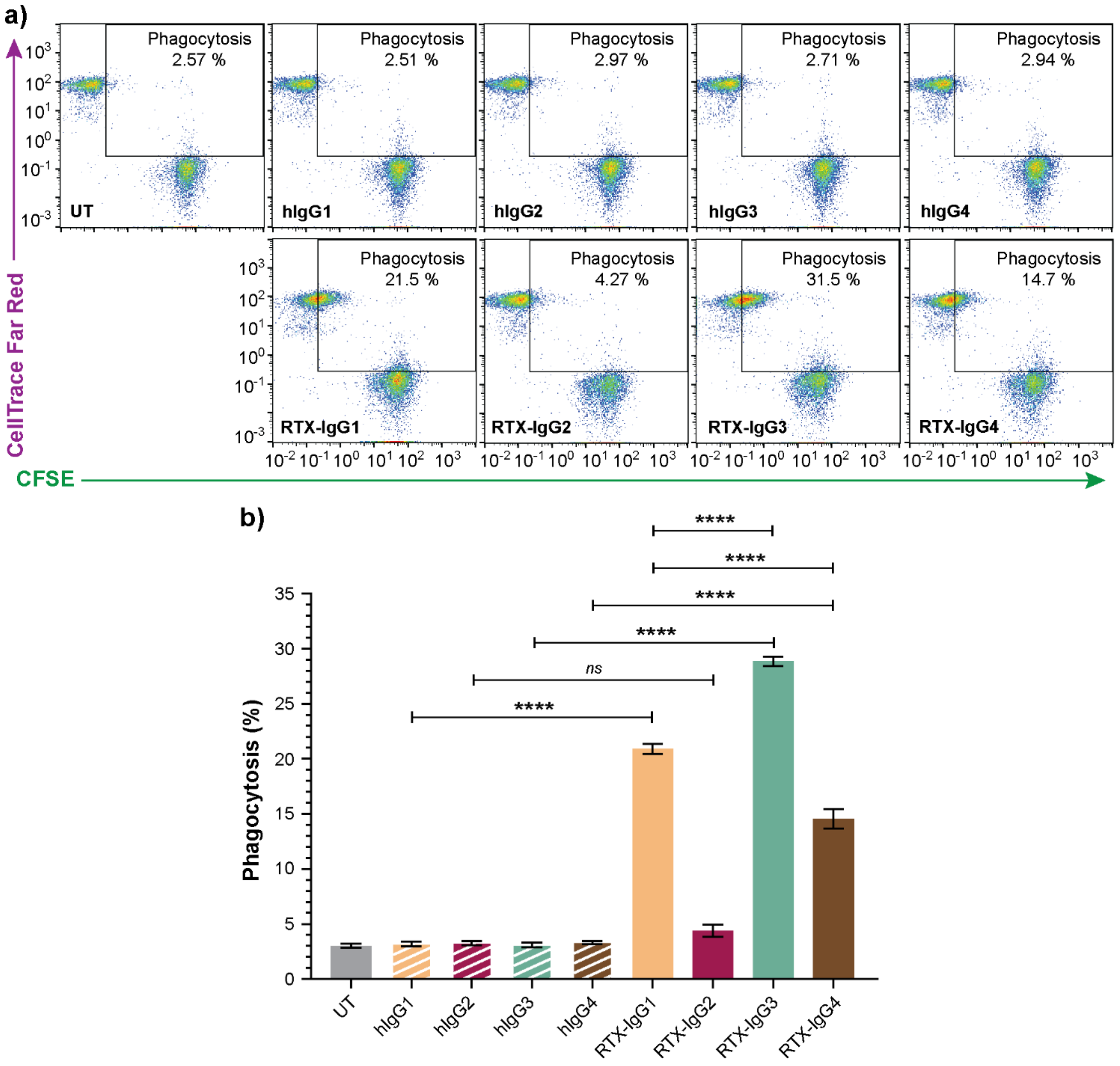

Figure S3. a) Representative flow plots showing ADCP of Granta-519 cells treated with RTX isotypes (RTX-IgG1-4) or isotype control Abs (hIgG1-4), by MonoMac-6 cells (E:T ratio = 1:1). The phagocytosis of CFSE-labeled Granta-519 cells was quantified as the percentage of double positive $CFSE^{+}$ $CTFR^{+}$ MonoMac-6 cells (rectangular gate). The gating was set based on unstained and single-colored stained controls. b) Percentage phagocytosis of Granta-519 cells, induced by single RTX-isotypes (IgG1-4) or human isotype controls (hIgG1-4), by MonoMac-6 cells (E:T ratio = 1:1). Untreated cells (UT) were used as controls. Results are shown as mean ± SEM of three independent experiments, each with three biological replicates. Statistical analysis by one-way ANOVA with Tukey-Kramer post-hoc test (****: $p<0.0001$, ns = not significant).

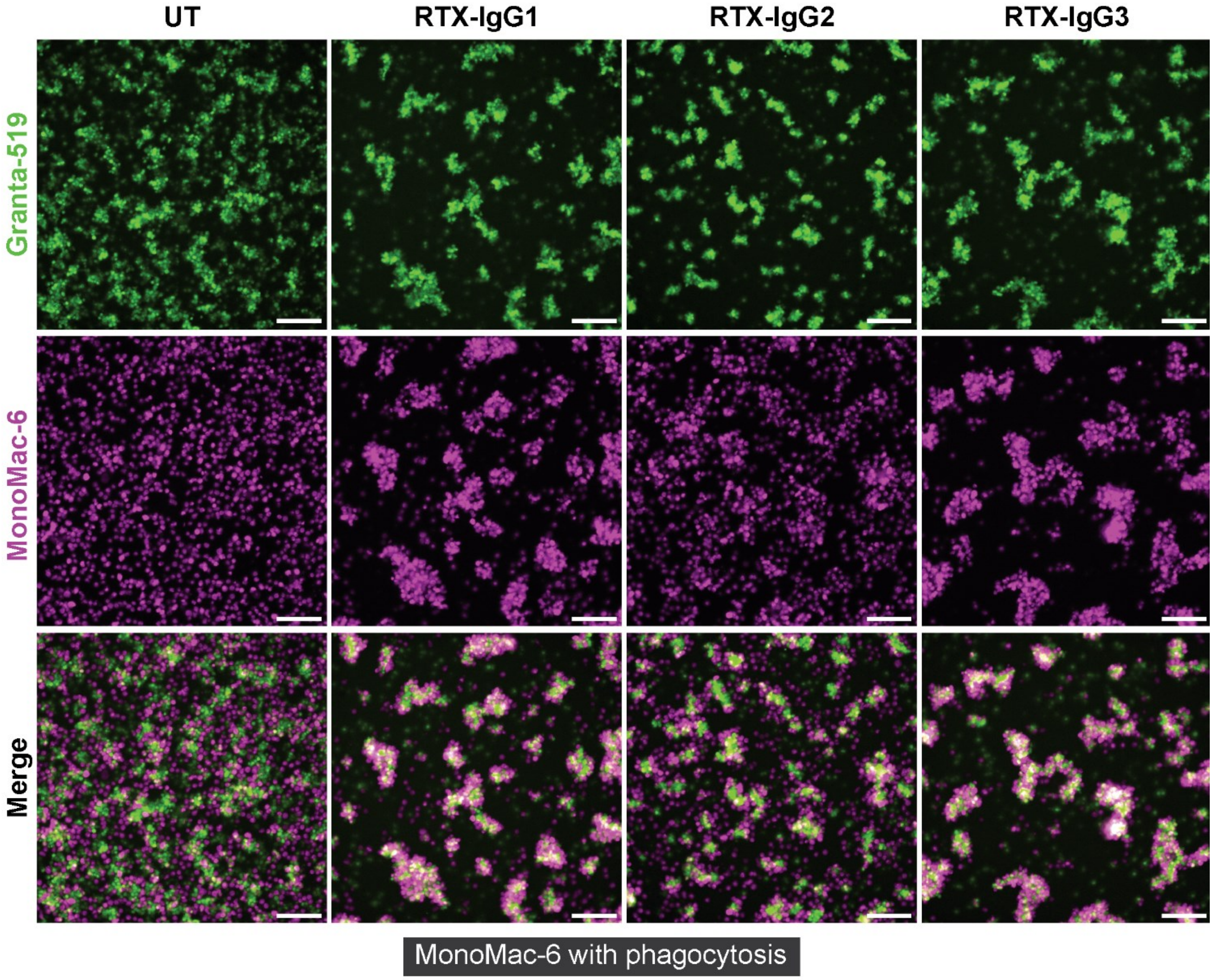

Figure S4. Microscopic analysis of ADCP of CD20^{+} B-cell lymphoma cells (Granta-519), induced by RTX-IgG1, RTX-IgG2, or RTX-IgG3 by MonoMac-6 cells (E:T ratio = 1:1). All tested RTX isotypes induced a comparable level of homotypic adhesion in CFSE-labeled Granta-519 cells (top panel). The majority of CTFR-labeled MonoMac-6 were attracted to the vicinity or attached to Granta-519 cell clusters in the presence of RTX-IgG1 or RTX-IgG3 (middle panel), with many MonoMac-6 cells becoming double positive for CFSE and CTFR (white color, bottom panel), indicating efficient phagocytosis. In contrast, significantly less MonoMac-6 cells attached to RTX-IgG2-treated Granta-519 cell clusters (middle panel). Only a few CFSE^{+} CTFR^{+} MonoMac-6 cells were observed in untreated (UT) or RTX-IgG2-treated co-cultures (bottom panel). Scale bars: 200μm.

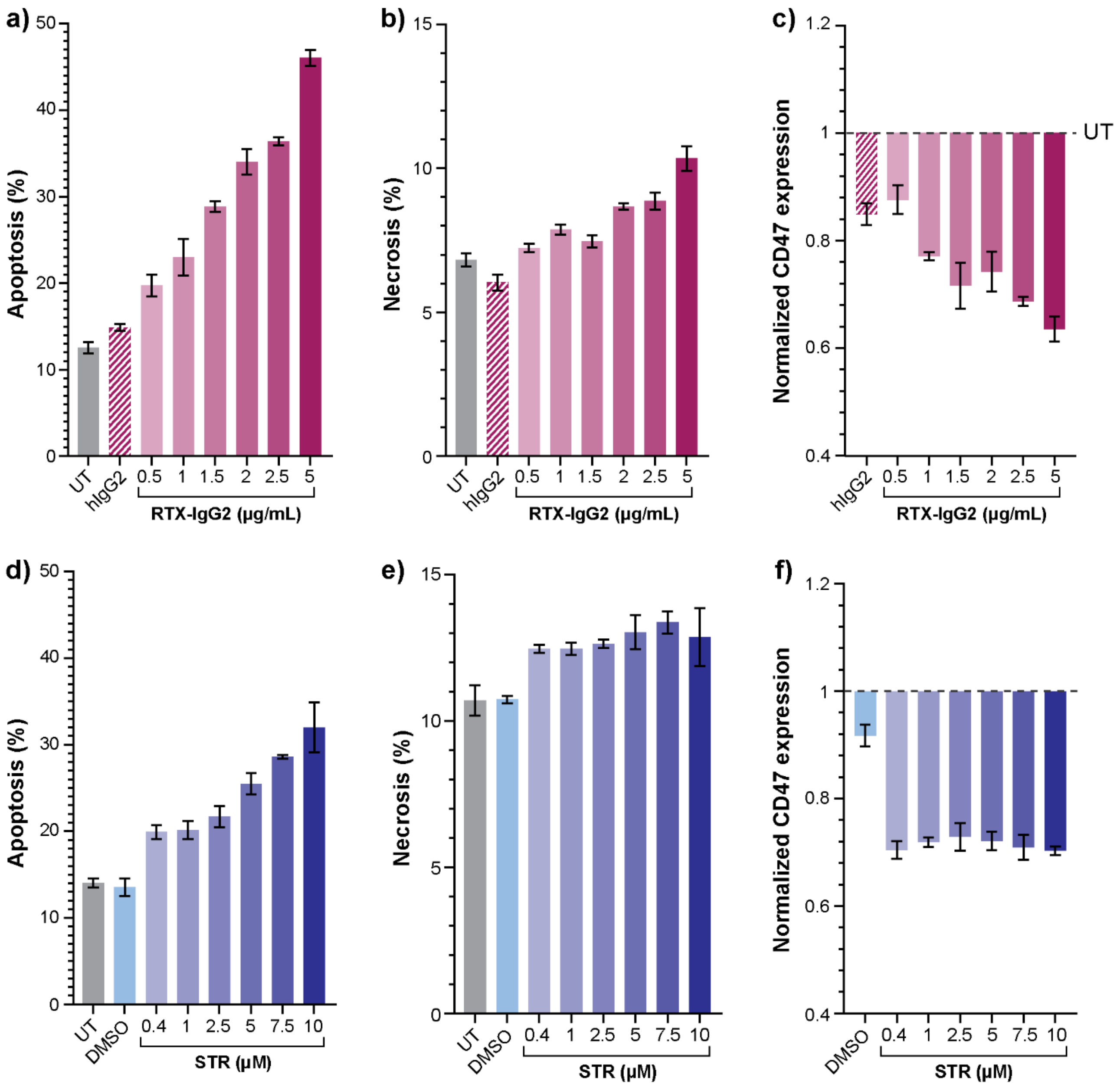

Figure S5. Analysis of apoptosis, necrosis and CD47 expression in Granta-519 cells treated with different concentration of a-c) RTX-IgG2 for 30 min or d-f) STR for 6 hours before analysis. Data are presented as mean ± SEM of three biological replicates. Untreated cells (UT) and hIgG2 isotype control Ab were used as negative controls, while DMSO was used as vehicle control of STR treatment.

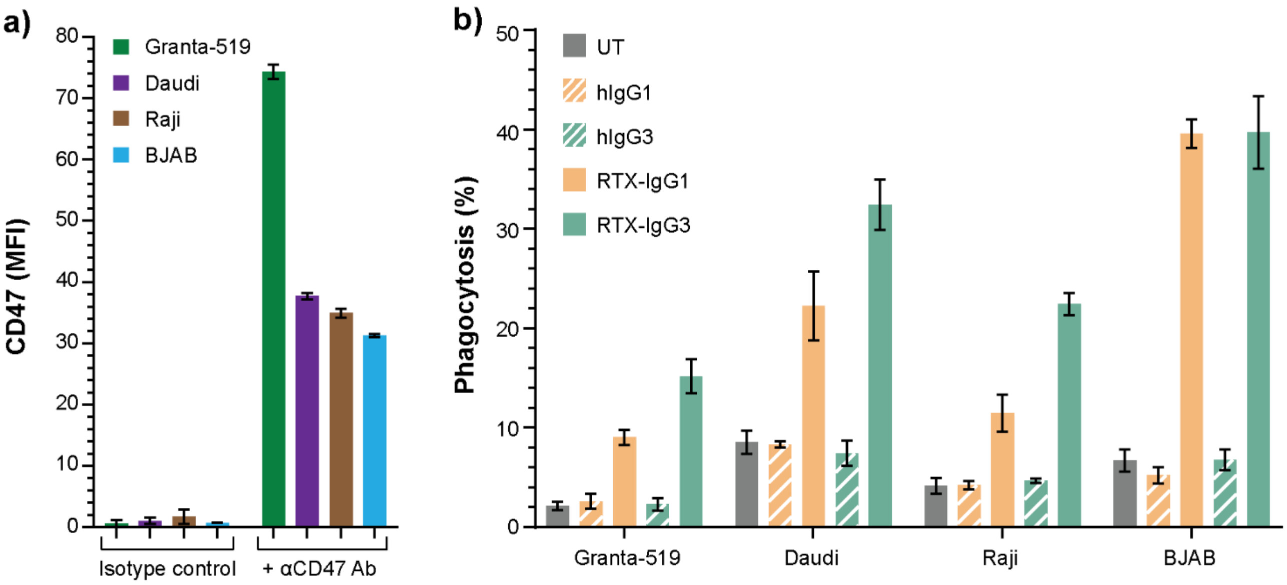

Figure S6. a) CD47 expression in four CD20^{+} B-cell lymphoma cell lines. b) RTX-IgG1 and RTX-IgG3-mediated ADCP in different B-cell lymphoma cell lines by MonoMac-6 effector cells (E:T ratio = 1:1). Data are presented as mean ± SEM of three biological replicates.

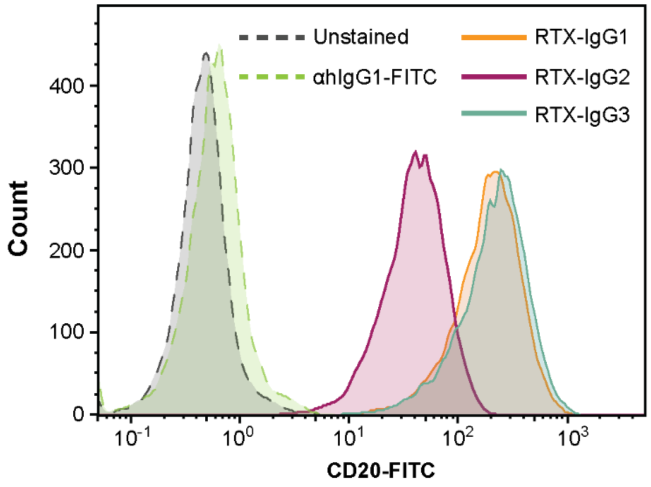

Figure S7. Binding of RTX-IgG1, RTX-IgG2, and RTX-IgG3 to CD20 on Granta-519 cells. Cells were incubated with RTX for 30 min at 37°C and subsequently stained with FITC-conjugated anti-human IgG Ab (αhIgG1-FITC).
